# Hybrid reflection retrieval method for terahertz dielectric imaging of human bone

**DOI:** 10.1364/BOE.427648

**Published:** 2021-07-12

**Authors:** Suzanna Freer, Cong Sui, Stephen M. Hanham, Liam M. Grover, Miguel Navarro-Cía

**Affiliations:** 1 School of Physics and Astronomy, University of Birmingham, Birmingham B15 2TT, UK; 2 School of Chemical Engineering, University of Birmingham, Birmingham B15 2TT, UK; 3 Department of Electronic, Electrical and Systems Engineering, University of Birmingham, Birmingham B15 2TT, UK

## Abstract

Terahertz imaging is becoming a biological imaging modality in its own right, alongside the more mature infrared and X-ray techniques. Nevertheless, extraction of hyperspectral, biometric information of samples is limited by experimental challenges. Terahertz time domain spectroscopy reflection measurements demand highly precise alignment and suffer from limitations of the sample thickness. In this work, a novel hybrid Kramers-Kronig and Fabry-Pérot based algorithm has been developed to overcome these challenges. While its application is demonstrated through dielectric retrieval of glass-backed human bone slices for prospective characterisation of metastatic defects or osteoporosis, the generality of the algorithm offers itself to wider application towards biological materials.

## 1. Introduction

Terahertz radiation grants us access to a unique view of the world. Superior resolution over microwave imaging, alongside non-ionising effects compared to X-rays, has resulted in the exploitation of terahertz radiation for applications [1] such as security screening [2,3] and quality control [4]. Particular interest has been focused on biological imaging [5], where terahertz energies match low frequency vibrations of biomolecules [6] and rotational modes of liquid water molecules, which can be utilised for sensing and imaging [7–9]. Such applications include characterisation of human bone metastatic defects or osteoporosis and can be extended to prostheses design [10–13]. The development of this powerful technique, however, remains limited by challenges in extraction of meaningful biometric information from measurements.

The typically high water content of biological materials, alongside its highly absorbing properties at terahertz frequencies, make reflection spectroscopic measurements far more practical than transmission. Accurate retrieval of both the amplitude and phase of the reflected radiation relies on a number of factors. The two critical factors, for time domain spectroscopy (TDS) in particular, are accurate alignment of the sample and reference planes [14,15] and the ability to resolve or model any Fabry-Pérot reflections that occur within the sample [16–19]. Here, we develop a hybrid retrieval algorithm to account for possible experimental misalignment and unresolvable reflections in thin samples through application of the Kramers-Kronig relations and modelling Fabry-Pérot effects. Our methodology improves upon existing algorithms incorporating Fabry-Pérot modelling for reflection measurements [16–19] by developing a self-reliant method of estimating sample thickness, without the need of an initial measurement and reducing computational complexity through two-dimensional scanning of dielectric properties in the frequency domain. Additionally, the generality of the approach means that its application is not limited by material thickness, absorption and reflectivity. Application of the algorithm is demonstrated on glass-backed heterotopic ossification (HO) human bone slices for prospective characterisation of disease. Needless to say, the algorithm can be applied for wider range of applications, but this is not within the scope of this paper.

## 2. Methods

### 2.1 Kramers-Kronig phase retrieval

TDS in reflection geometry is a common technique used to measure the interaction of a terahertz electric field with a sample upon reflection. Depending on the dielectric environment of the sample, the amplitude and phase of the field is modified. Practically, retrieval of the phase is challenging. Phase is highly sensitive to the sample position on the micron scale [20,21]. Any offset between a reference and sample plane ΔL induces an accumulated phase offset of Δθ(ω)=ωn(2ΔL/c), where n is the refractive index of air and ω and c are the angular frequency and vacuum speed of the wave, respectively. Difficulties in precise positioning, despite experimental efforts [20–22], mean researchers have turned to post processing numerical methods to overcome this challenge, with particular focus on the principle of causality and its expression in the Kramers-Kronig relations [14,23,24].

The Kramers-Kronig relations are mathematical bidirectional equations that describe a fundamental relation between the real and imaginary parts of a linear complex function, for which the principle of causality stands. That is, the response of a system is zero until an impulse is applied to it. The refractive index of a material is a complex function which can be expressed in this way, where the real and imaginary parts describe the energy storage due to material polarisation and absorption, respectively. Hence, from measurements of the dispersion of a field propagating through a material, one can obtain the absorption, and vice versa. This fundamental relationship has found wide application in linear [25–27], and subsequently non-linear, optics [28–30]. A limitation of the Kramers-Kronig relations is that the knowledge of the spectrum must be across a semi-infinite frequency range. This limitation can be relaxed through application of Singly Subtractive Kramers-Kronig (SSKK) relations, for improved convergence [29–31].

This work implements an algorithm developed by V. Lucarini and colleagues [30], through application of SSKK relations to the real and imaginary parts of the reflected field defined as r(ω)=|r(ω)|exp⁡(iθ(ω)), or its logarithmic amplitude ln⁡|r(ω)| and phase θ(ω). The dispersion integrals are given by

(1) $$\ln|r(\omega )|-\ln|r(\omega_{1})|=\frac{2(\omega^{2}-\omega_{1}^{2})}{\pi }P\int_{0}^{\infty }\frac{\omega^{\prime }\theta (\omega^{\prime })}{\left(\omega^{\prime 2}-\omega^{2})(\omega^{\prime 2}-\omega_{1}^{2}\right)}\;d\omega^{\prime },$$

(2) $$\frac{\theta (\omega )}{\omega }-\frac{\theta (\omega_{1})}{\omega_{1}}=\frac{2(\omega^{2}-\omega_{1}^{2})}{\pi }P\int_{0}^{\infty }\frac{\ln|r(\omega^{\prime })|}{\left(\omega^{\prime 2}-\omega^{2})(\omega^{\prime 2}-\omega_{1}^{2}\right)}\;d\omega^{\prime },$$

where r(ω) is the complex reflection coefficient, $\omega_{1}$ is a frequency anchor point, P is the Cauchy principle value and θ(ω) is the phase. The inability to retrieve the phase from the amplitude, and vice versa, indicates an error in the measurements. An optimisation approach can be applied to obtain this error, and hence, the phase offset.

**Fig. 1.**
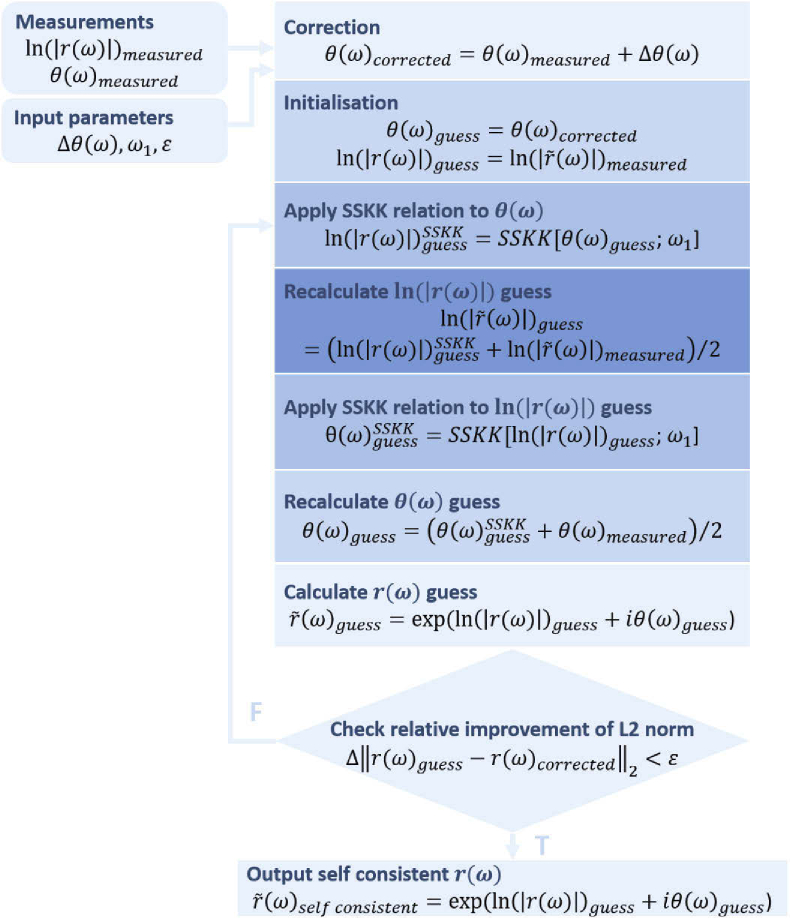
Flow diagram of the Kramers-Kronig phase retrieval algorithm.

The optimisation approach, presented in Fig. 1, involves the iterative application of the SSKK relations to the amplitude ln⁡|r(ω)| and a corrected phase $\theta (\omega )=\theta {\left(\omega \right)}_{measured}+\Delta \theta (\omega )$ for a range of guessed phase offsets Δθ(ω). For each iteration, the degree of consistency between the two relations is calculated using the L2 norm $\|r{\left(\omega \right)}_{guess}-r{\left(\omega \right)}_{corrected}{\|}_{2}$. The application is repeated until the consistency has an incremental improvement less than ϵ (=0.01). Subsequently, the self-consistent reflectivity is calculated, from which the phase offset is extracted. Convergence is typically achieved after 10 iterations.

**Fig. 2.**
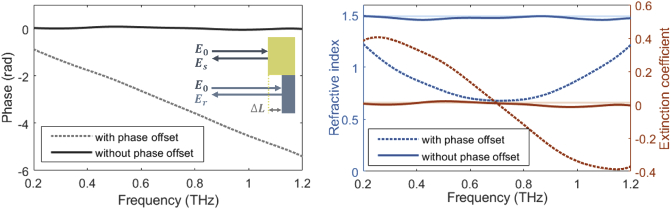
Phase response (left) and extracted dielectric properties (right) of paraffin wax obtained with a sample-reference plane offset of ∼200 μm, before and after the Kramers-Kronig retrieval algorithm is applied. The transparent lines in the dielectric results indicate the literature values [19]. The inset shows an illustration of the offset of the reference plane to the sample plane. $E_{0}$, $E_{s}$ and $E_{r}$ are the incident, sample and reference electric field amplitudes, respectively.

Figure 2 demonstrates the application of the algorithm to TDS measurements of paraffin wax with an offset of ∼ 200 μm between the sample and reference planes. Prior to the phase offset retrieval, one can observe a large change in phase with frequency in the left panel, attributed to the additional phase acquired over distance ΔL. This results in highly distorted extracted dielectric properties, presented in the right panel. The corrected phase acquired from the algorithm is shown to have a much lower frequency dependence, enabling reliable extraction of the refractive index and extinction coefficient, consistent with values reported in the literature [19], illustrated by the transparent lines in Fig. 2(right).

The extracted phase offset was found to be robust, independent of the anchor frequency selection, given that they are within the measured frequency range. The uncertainty of the output phase is largely dependent on the estimated phase offset increment, while the run time of the algorithm is limited by both the number of input estimated offsets and anchor frequencies. Calculations of errors of the Kramers-Kronig algorithm can be found in Ref. [23]. It should also be noted that while the algorithm is applied to cases of normal incidence, its application can be extended to oblique incidence, accounting for the additional path length travelled by the field due to the additional angle θ, where the effective path length is given by $L_{\text{eff}}=L/\cos\theta$.

For the case of optically thick samples, the fundamental causal basis of the Kramers-Kronig retrieval algorithm means it can be used for reliable extraction of dielectric properties for a vast range of samples, without prior knowledge of the dielectric response. For optically thin samples, however, where pulses overlap, Fabry-Pérot reflections come into play.

### 2.2 Fabry-Pérot dielectric property retrieval

Fabry-Pérot reflections of electromagnetic fields occur in cavities made from two parallel reflecting surfaces formed from refractive index boundaries. They are often exploited in spectroscopy to increase field-sample interactions. For TDS measurements of thin samples, however, they can become problematic. For pulsed excitation, individual reflected pulses will exit the cavity at different times depending on the cavity thickness. For thicker cavities, the reflections will be spread out further in time. The ability to resolve the reflected pulses can be crucial in TDS for reliable dielectric property extraction. It is worth noting that difficulties in pulse resolution extend to highly dispersive materials, where part of the pulse may overlap itself after a reflection.

**Fig. 3.**
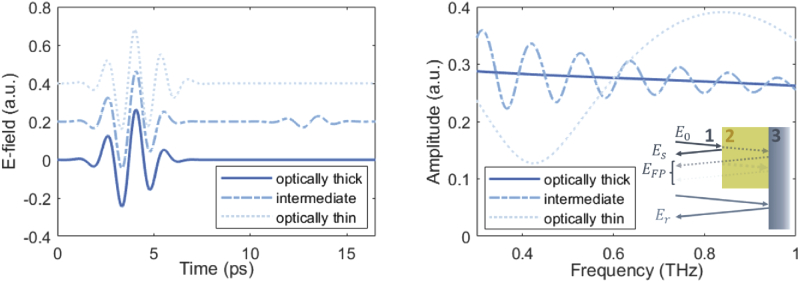
Time domain (left) and frequency domain (right) response of optically thick, thin and intermediate structures under normal incidence, simulated using the transient solver of CST Microwave Studio. Layer 1 was modelled as air, while layers 2 and 3 were modelled as bone and glass, with refractive index values of 1.8 and 2.6 and extinction coefficient values of 0.08 and 0.15, respectively. The temporal waveforms are offset along the y-axis with respect to each other by 0.2 a.u. for clarity. The inset illustrates the sample configuration, for which the sample (layer 2) sits on a reference layer (layer 3). $E_{0}$, $E_{s}$, $E_{r}$ and $E_{\text{FP}}$ are the incident, sample, reference and Fabry-Pérot electric fields, respectively.

Figure 3 demonstrates the effect of Fabry-Pérot reflections on the retrieved spectral response of the material under normal incidence conditions. The reflected time and frequency responses were simulated using the transient solver of CST Microwave Studio, which uses the finite integration technique to solve Maxwell’s equations, for structures (illustrated in the inset) of different thicknesses. The structures were designed to have dielectric properties comparable to the glass-backed HO bone structures the algorithm is subsequently applied to. For the optically thick sample, no Fabry-Pérot reflections are measured, since the first reflection occurs after the measurement window, and hence the true spectral response is extracted. When reflections are recorded in the waveform by reducing the thickness of the sample, oscillations emerge in the spectral response, with decreasing frequency for decreasing thicknesses. This, in turn, induces artifacts in the extracted dielectric properties.

For the intermediate case, the Fabry-Pérot reflection at ∼13 ps can easily be resolved from the initial reflection, and hence the resultant artifacts removed by standard time-windowing. For the optically thin sample, however, the initial and subsequent reflections overlap completely. Separation of the terahertz pulses is a well known challenge in the TDS terahertz community for transmission measurement, where researchers have focused efforts on overcoming the problem through methods such as modelling the reflections [16,18,32–35]. These methods can be translated to overcome the same challenges presented in reflection measurements.

Here, an algorithm based on frequency domain modelling of the reflected field from TDS measurements alternative to those reported in the literature [16,18,32–35] is presented. The algorithm offers reduced computational complexity and relaxation of limits in sample thickness, absorption and reflectivity. The field is assumed to be a plane wave with linear polarisation. As mentioned, to extract the dielectric properties of the sample under investigation, TDS measurements typically involve a measurement of the reflected field for both the reference sample with known dielectric response and the investigated sample. Here, as illustrated in the inset of Fig. 3, the field is modelled for a thin sample (layer 2) fixed to a reference sample (layer 3). The versatility of this approach means that it can be applied to a range of configurations through appropriate field modelling. From the Fresnel coefficients, the sample and reference fields are given by

(3) $$E_{ref}(\omega )=P_{1}^{2}R_{13}E_{0},$$

(4) $$E_{samp}(\omega )=A_{wo,1}R_{12}E_{0}+\underset{\text{Fabry-Perot terms}}{\underbrace{\{A_{wo,2}T_{12}P_{2}^{2}R_{23}T_{21}+A_{wo,3}T_{12}P_{2}^{4}R_{23}^{2}R_{21}T_{21}+\cdots \}E_{0}}},$$

where ω is the angular frequency, $E_{0}$ is the incident electric field, R, T and P are the reflection, transmission and propagation coefficients, and $A_{wo,m}$ is a frequency- and angle-of-incidence-dependent complex factor that takes into account the walk-off effect of the Fabry-Pérot etalons that results in a different coupling of each reflected sample signal to the detector compared to the reference signal. This factor is difficult to quantified formally, but it can be estimated empirically by calibration [36]. For very thin samples (i.e. layer 2), $A_{wo,m}\approx 1$ and for normal illumination $A_{wo,m}=1$. The reflection coefficients for each polarisation (s/p) of the incident field are given by

(5) $$R_{ij,s}(\omega )=\frac{{\tilde{n}}_{i}\cos\theta_{i}-{\tilde{n}}_{j}\sqrt{1-{\left(\frac{{\tilde{n}}_{i}}{{\tilde{n}}_{j}}\sin\theta_{i}\right)}^{2}}}{{\tilde{n}}_{i}\cos\theta_{i}+{\tilde{n}}_{j}\sqrt{1-{\left(\frac{{\tilde{n}}_{i}}{{\tilde{n}}_{j}}\sin\theta_{i}\right)}^{2}}},$$

(6) $$R_{ij,p}(\omega )=\frac{{\tilde{n}}_{i}\sqrt{1-{\left(\frac{{\tilde{n}}_{i}}{{\tilde{n}}_{j}}\sin\theta_{i}\right)}^{2}}-{\tilde{n}}_{j}\cos\theta_{i}}{{\tilde{n}}_{i}\sqrt{1-{\left(\frac{{\tilde{n}}_{i}}{{\tilde{n}}_{j}}\sin\theta_{i}\right)}^{2}}+{\tilde{n}}_{j}\cos\theta_{i}},$$

with (ij)= (12), (13), (23) and (21), where 1, 2 and 3 are material labels, and $\tilde{n}$ is the complex refractive index. The transmission coefficients are related to the reflection coefficients as

(7) $$T_{kl,s}(\omega )=1+R_{kl,s},$$

(8) $$T_{kl,p}(\omega )=(1+R_{kl,p})\frac{\cos\theta_{1}}{\cos\theta_{2}},$$

with (kl)= (12) and (21), where $\theta_{2}$ is the angle of refraction and incidence inside material 2, and is defined by $\theta_{2}=\sin^{-1}\left(\left({\tilde{n}}_{1}/{\tilde{n}}_{2}\right)\sin\theta_{1}\right)$. The propagation coefficients is given by

(9) $$P_{q}(\omega ,L)=\exp\left(-i{\tilde{n}}_{q}\omega (L/\cos\theta_{q})/c\right),$$

where q is the material label for layers 1 and 2. The transfer function, defined as the ratio of the sample and reference fields, is therefore given by

(10) $$H(\omega )=\frac{1}{P_{1}^{2}R_{13}}[A_{wo,1}R_{12}+\underset{\text{Fabry-Perot terms}}{\underbrace{\left\{A_{wo,2}T_{12}P_{2}^{2}R_{23}T_{21}+A_{wo,3}T_{12}P_{2}^{4}R_{23}^{2}R_{21}T_{21}+\cdots \right\}}}].$$

Providing the dielectric response of the reference material is known, the properties of the sample can be retrieved through a comparative approach. Here, medium 3 is thick enough such that Fabry-Pérot reflections are outside the measurement window and hence the terms within the layer do not need to be considered. If the layer was thin enough, however, the reflections could be incorporated.

**Fig. 4.**
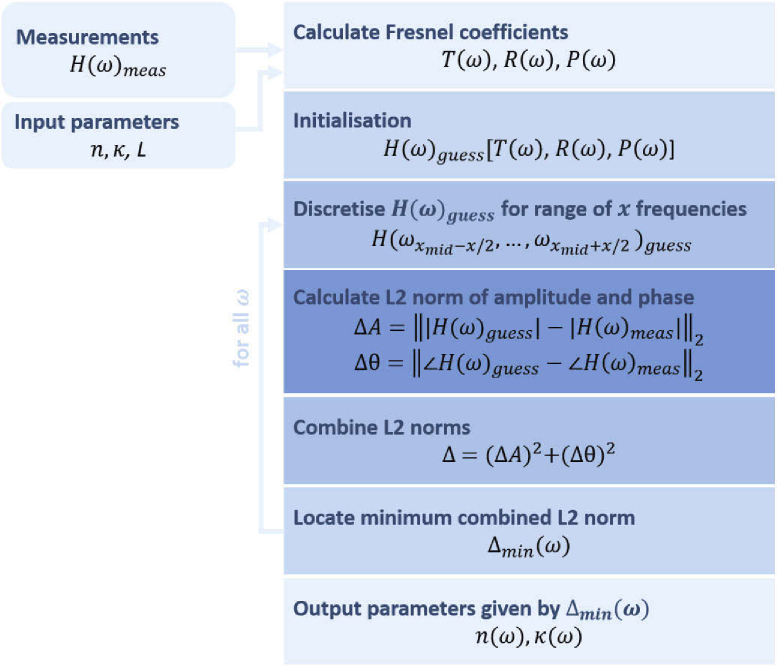
Flow diagram of the Fabry-Pérot dielectric extraction algorithm.

The algorithm, presented in Fig. 4, utilises a frequency dependent two-dimensional scanning approach of a parameter space, involving the calculation of the transfer function $H{\left(\omega \right)}_{guess}$ for a range of estimates of the refractive index n and extinction coefficient κ of the sample. The sample thickness L is given by the phase correction from the Kramers-Kronig algorithm. The transfer function is then discretised with respect to the frequency and the error between the measured and estimated transfer functions, $H{\left(\omega \right)}_{meas}$ and $H{\left(\omega \right)}_{guess}$, is then calculated using the combined L2 norm of the amplitude and phase. The output values of the refractive index and extinction coefficient are given by the minimum error, as a function of frequency.

While the simplicity of the approach enables straightforward implementation for applications, there exists limitations of the algorithm. The minimum detectable thickness, for example, is naturally system dependent, since it depends on the pulse duration. CST Microwave Studio was used to study the thickness limit through application of the Fabry-Pérot algorithm to the reflected field, for decreasing structure thicknesses. A 0.3 – 1 THz Gaussian pulse was used. The algorithm was able to successfully model the reflections until thicknesses reached as low as 0.1 μm. Uncertainty of the extracted values depends on the increments of the parametric sweep of dielectric properties. Additional uncertainty stems from the discretisation of the transfer function with respect to frequency, and in turn, the frequency resolution. Specifically for Gaussian beam propagation as in THz TDS systems and oblique incidence, an uncertainty in path length will be caused by the finite width of the Gaussian beam, that is not taken into account in the above derivation based on geometrical optics. All these factors affect computational run time, and hence compromises must be made depending on the application.

**Fig. 5.**
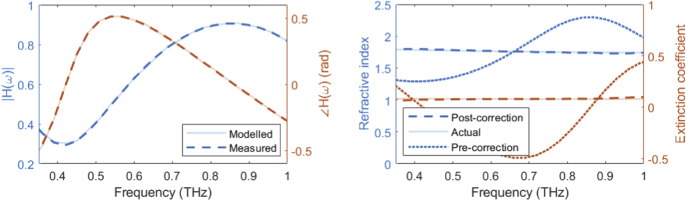
(Left) Amplitude and phase of the transfer function H(ω) for the structure presented in Fig. 3, simulated using the transient solver of CST Microwave Studio. Layer 1 was modelled as air, while layers 2 and 3 were modelled as bone and glass, respectively. The dashed lines illustrate the measured transfer function, while the transparent lines illustrate the transfer function modelled using the Fabry-Pérot algorithm. (Right) Refractive index and extinction coefficient of the structure pre- and post-application of the Fabry-Pérot modelling algorithm, indicated by the dotted and dashed lines, respectively. The transparent lines illustrate the input dielectric properties in CST Microwave Studio.

Figure 5 presents the reflected transfer function for normal incidence (i.e. $\theta_{1}=\theta_{2}=0$, and $A_{wo,m}=1$) for a 100 μm structure, pre- and post-application of the Fabry-Pérot modelling algorithm. The temporal waveforms were simulated using the transient solver of CST Microwave Studio and the structure was designed to have dielectric properties comparable to the glass-backed HO bone (see sample configuration in the inset of Fig. 3). One can observe low frequency oscillations attributed to the Fabry-Pérot reflections within the structure. The algorithm successfully models the reflections, providing good agreement between the measured and modelled transfer function. This enables the removal of these oscillating artefacts, which propagate through to the refractive index and extinction coefficient results. The right panel of Fig. 5 presents the corrected results, demonstrating the successful extraction of the correct dielectric properties.

It is important to note that the phase sensitivity of the dielectric response means that the performance of the Kramers-Kronig algorithm prior to Fabry-Pérot modelling is crucial for reliable dielectric properties retrieval. For the given sample setup, by applying the Kramers-Kronig algorithm, the plane of the reference reflection is essentially brought forward to the sample plane. The propagation $P_{1}$ is therefore equal to unity.

## 3. Results and discussion

**Fig. 6.**
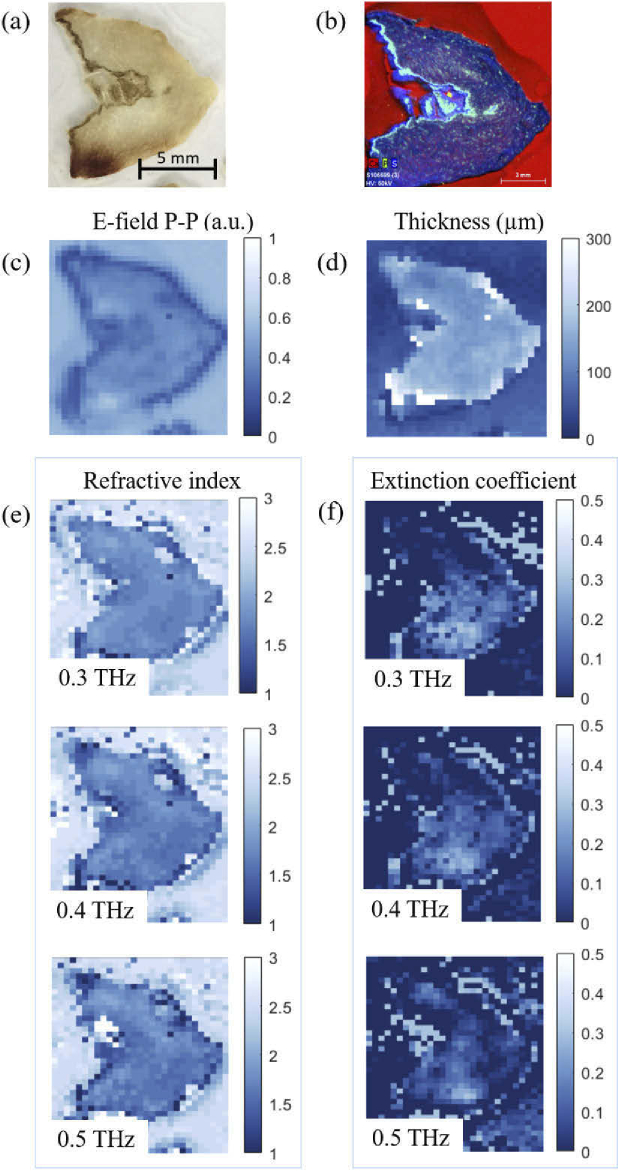
(a) Photograph of a heterotopic ossification (HO) bone slice embedded in resin and fixed to a glass slide. (b) XRF image, indicating the elemental distributions of the bone slice, where calcium, phosphorus and sulphur (representing collagen) are represented by red, green and blue, respectively. The yellow regions indicate the colocalisation of calcium and phosphorus (representing CaP). Images of the (c) peak-to-peak value of the temporal reflected electric field, (d) extracted sample thickness. Normal incidence images of the extracted (e) refractive index and (f) extinction coefficient values at 0.3 THz, 0.4 THz and 0.5 THz, post application of the retrieval algorithm.

To demonstrate the performance of the hybrid algorithm for reliable dielectric imaging, it is applied to TDS images of optically thin biological samples which exhibit an offset between the sample and reference planes. The structures are ∼100 μm thick HO bone slices, embedded in resin and fixed to a glass slide, as photographed in Fig. 6(a) (see Appendix A for details). The normal incidence measurements were taken in reflection geometry through single pixel raster scan imaging. The peak-to-peak value of the temporal E-field is presented in Fig. 6. The reference measurement was taken from a glass slide pixel, which sits further back from the bone (see the inset of Fig. 3), inducing a phase offset, while the thickness of the slices mean that the initial and subsequent reflections are unresolvable.

**Fig. 7.**
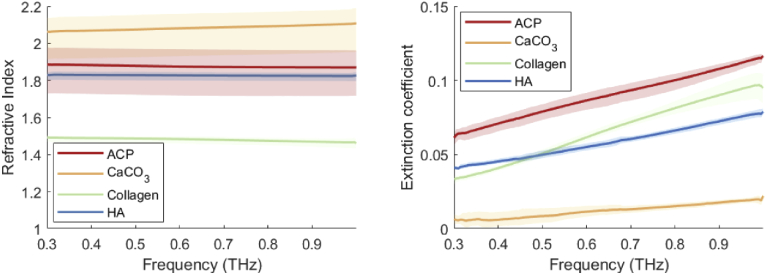
The refractive index and extinction coefficient of constituent compounds of bone structures: ACP, ${\mathrm{CaCO}}_{3}$, collagen and HA, measured using terahertz transmission TDS.

It is worth noting that for these samples in particular, no spectral fingerprints are expected in the probing frequency range. Spectral measurements of the constituent compounds of bone structures, including collagen and HA, are presented in Fig. 7 in Appendix B. The responses reveal reasonably featureless dielectric properties in this regime, for all compounds. Identification of compounds therefore relies on broadband differences in the refractive index and extinction coefficient.

**Fig. 8.**
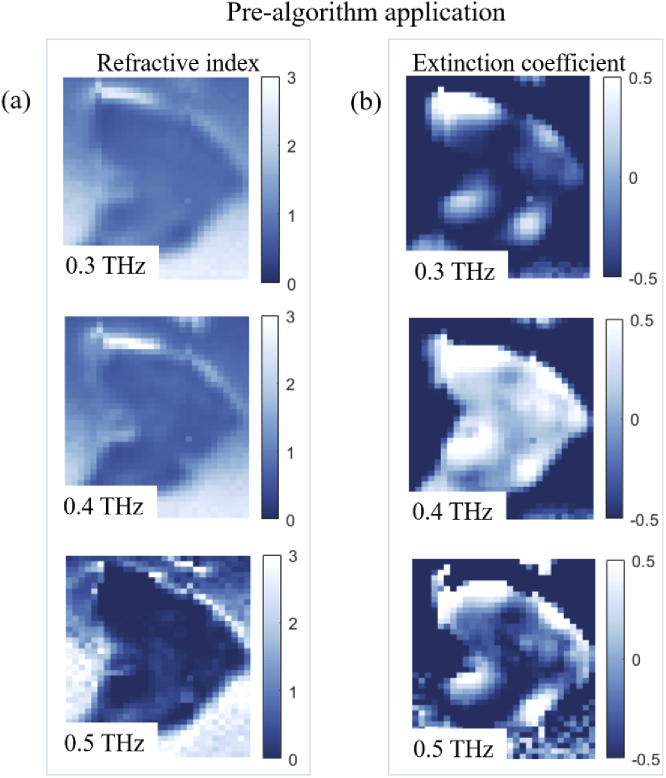
Images of the refractive index and extinction coefficient calculated from TDS reflections measurements of glass-backed HO bone slices, without application of the hybrid algorithm, at frequencies of 0.3 THz, 0.4 THz and 0.5 THz. Note the limits of the colour bar.

**Fig. 9.**
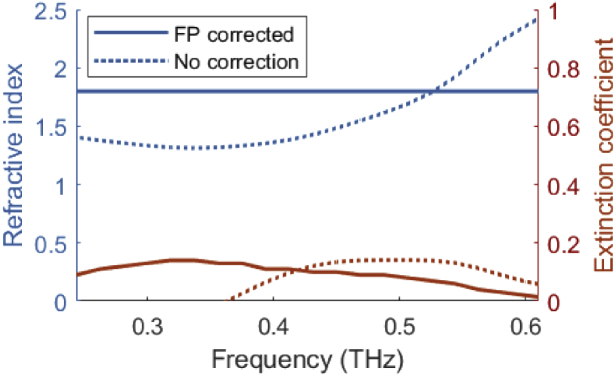
Refractive index and extinction coefficient of a ∼100 μm thick glass-backed HO bone sample pre- and post-application of the Fabry-Pérot modelling algorithm.

Images of the dielectric properties of the structure calculated prior to the application of the hybrid algorithm are presented in Fig. 8 in Appendix C, at 0.3 THz, 0.4 THz and 0.5 THz. The images display unphysical dielectric properties, with refractive indices of less than one and negative extinction coefficients. These are attributed to both the phase offset between the bone and glass planes and the Fabry-Pérot reflections supported by the bone cavity. To demonstrate the performance of the hybrid algorithm for a single pixel, Fig. 9 (Appendix C) presents the hyperspectral dielectric properties of the structure pre- and post-application. One can observe the removal of the oscillating artifacts, attributed to phase accumulation and Fabry-Pérot reflections simultaneously, to reveal dielectric properties with low frequency dependence.

Figure 6 presents the imaged dielectric properties of the bone slice subsequent to the application of the algorithm. The composition of the bone sample is revealed in the XRF image in Fig. 6(b). The image identifies the bulk of the sample as collagen (blue), with small islands of hydroxyapatite (HA), a form of calcium phosphate (yellow). From measurements of the dielectric properties of the constituent compounds of bone structures presented in Fig. 7 in Appendix B, collagen is found to have a refractive index of ∼1.5, with low frequency dependence, and an extinction coefficient of ∼0.04 – 0.09, from 0.3 – 1 THz. The small deviation from the retrieved values of ∼1.8 and ∼0.1 for the refractive index and extinction coefficient respectively, is thought to be attributed to an increased hydration level in the bone structures, to which terahertz is highly sensitive to. Water represents up to 25% of bone [37]. Although room-temperature dehydrated bone samples lose most of the bulk water and surface water layer, strongly bound and structural water remains [38]. Indeed, even after a ${\mathrm{225}}^{\circ }$C drying process, ∼40% of such tightly bound and structural water remains [38]. Hence, structural water is expected in the bone samples, while the collagen samples are dehydrated [39]. These findings demonstrate the success of the algorithm for dielectric extraction, with vast improvement upon the dielectric properties extracted prior to application.

It should be noted that the regions of HA are undetectable in the terahertz images due to large scattering of the field at the edges of the structure and low resolution. The resolution could be improved through excitation with higher frequencies. The dielectric images demonstrate marginal improvement upon resolution from 0.3 THz to 0.5 THz, however, pushing to higher frequencies would be beneficial. Unfortunately, the TDS system in use is limited at high frequencies due to low signal-to-noise ratio.

The dielectric images demonstrate the success of the hybrid algorithm for biometric imaging and its ability to deal with complex sample configurations. This is essential for practical biological application where controllability is limited. It should be noted that the free-standing sample set-up (i.e. not sandwiched between two windows, as commonly configured [19]), while making the system far more versatile, means that results are affected by surface roughness and resultant scattering [40]. This should be considered when dealing with structures of high roughness. The assumptions of plane wave excitation and a linear electromagnetic response of the sample should also be considered for application of the algorithm. The former assumption is important for TDS excitation where the divergence of the beam is large, particularly at low frequencies, and its profile deviates from a typical Gaussian distribution [41].

## 4. Conclusion

Application of this novel hybrid Kramers-Kronig and Fabry-Pérot based algorithm becomes highly valuable in the retrieval of biometric information of biological materials, where samples exhibit high terhertz absorption and sample placement and thickness are difficult to control. Its application has been demonstrated through retrieval of dielectric information of glass-backed HO human bone slices, for which bulk compound composition is identified. The causal basis of the Kramers-Kronig approach and lifted limitation of sample thickness attributed to the Fabry-Pérot approach grants great flexibility of measurements, and hence wide application to biological materials.

## Data Availability

Data underlying the results presented in this paper are not publicly available at this time but may be obtained from the authors upon reasonable request.

## Appendix A: Preparation of bone samples

Heterotopic ossification (HO) samples were obtained from consented patients undergoing routine excision surgery at University Hospital Birmingham in United Kingdom. The HO samples were frozen immediately after excision surgery and stored at −${\mathrm{80}}^{\circ }$. Samples were thawed at ambient temperature for 1 h before use, and then embedded in optimal cutting temperature compound (VWR Chemicals, Leuven, Belgium) under vacuum (20 kPa) using a Struers CitoVac (Struers Ltd, Rotheram, United Kingdom). The embedded samples were equilibrated to −${\mathrm{80}}^{\circ }$C after refreezing at −${\mathrm{80}}^{\circ }$C overnight, and sectioned into 100 μm sample slices using a Leica microtome with a tungsten carbide blade. The obtained sample slices were mounted on a glass microscope slide and dried in ambient conditions for 72 h. Tissue transfer and handling was conducted under approval of the National Research Ethics Service (15/NW/0079) and in accordance with the Human Tissue Act 2004.

## Appendix B: Dielectric properties of constituent compounds of bone

The dielectric properties of constituent compounds which make up bone structures were measured using transmission TDS. The compounds include amorphous calcium phosphate (ACP), calcium carbonate (${\mathrm{CaCO}}_{3}$), collagen and hydroxyapatite (HA). The compounds were in powdered form, and pressed into pellets.

**Synthesis of hydroxyapatite (HA):** A solution of ${\left({\mathrm{NH}}_{4}\right)}_{2}$${\mathrm{HPO}}_{4}$ (0.58 M) was prepared in deionised water (DIW, 125 ml). $\text{Ca}{\left({\text{NO}}_{3}\right)}_{2}\cdot 4{\text{H}}_{2}\text{O}$ solution (0.48 M) prepared in DIW (175 ml) was added dropwise into the prior solution under vigorous magnetic agitation at ambient temperature. Both of the solutions were adjusted to pH 11 prior to mixing, and the whole procedure proceeded in a sealed vessel. The white precipitate was harvested by centrifugation (4000 rpm, 1 min) and washed with DIW for 3 times. The resulting HA powder was obtained by freeze drying.

**Synthesis of amorphous calcium phosphate (ACP):**$\text{Ca}{\left({\text{NO}}_{3}\right)}_{2}\cdot 4{\text{H}}_{2}\text{O}$ (9.26 g) was dissolved in DIW (110 ml), containing ammonia solution (28 wt.%, 8 ml). Ammonium phosphate solution was prepared by dissolving ${\left({\mathrm{NH}}_{4}\right)}_{2}$${\mathrm{HPO}}_{4}$ (5.44 g) in DIW (260 ml) containing ammonia solution (28 wt.%, 8 ml). The prepared ammonium phosphate solution was poured into the prior calcium nitrate solution under vigorous magnetic agitation at ambient temperature. The white precipitate was immediately harvested by centrifugation (4000 rpm, 1 min) and washed with DIW for 3 times. The resulting ACP powder was obtained by freeze drying.

**Synthesis of calcite:** A ${\mathrm{Na}}_{2}$${\mathrm{CO}}_{3}$ solution (0.1 M, 100 ml) was rapidly poured into ${\mathrm{CaCl}}_{2}$ solutions (0.1 M, 100 ml) under intense magnetic agitation and kept stirring at 400 rpm for 1 day at ambient temperature. The white precipitate was obtained by centrifugation (4000 rpm, 1 min) and washed with DIW for 3 times. The resulting calcite powder was dried at ${\mathrm{65}}^{\circ }$C for 5 days.

**Preparation of sample pellets:** The synthesised HA, ACP, calcite and commercially-purchased collagen powder (0.2 g) were compressed uniaxially in a stainless steel die with an internal diameter of 13.0 mm (Specac, UK) at a force of 7 kN by a universal material testing machine (Z030, Zwick Roell, Germany), respectively.

**TDS settings:** Focused illumination and detection was achieved using TPX50 lenses with 50 mm effective focal length. The dielectric properties were extracted using the commercial software Teralyzer, as in [43].

## Appendix D: TDS bone measurement method

The samples were characterized using an all fiber-coupled THz time-domain spectrometer TERA K15 from Menlo Systems with lock-in detection. The time-constant was set to 300 μs. The temporal length of the waveforms was 52 ps, providing a spectral resolution of 15 GHz. A standard reflection configuration was setup with a Si beam-splitter. The field was normally incident with accuracy of 0.3 deg and focused on the sample using a TPX35 lens with 35 mm effective focal length. To generate the images, a single pixel imaging technique was used, through translation of the sample fixed to an xy-translation stage, with steps of 0.5 mm.
